# SUMO E3 Ligase PIASy Mediates High Glucose-Induced Activation of NF-*κ*B Inflammatory Signaling in Rat Mesangial Cells

**DOI:** 10.1155/2017/1685194

**Published:** 2017-09-05

**Authors:** Wei Huang, Yaling Liang, Jianhua Dong, Luping Zhou, Chenlin Gao, Chunxia Jiang, Meijuan Chen, Yang Long, Yong Xu

**Affiliations:** ^1^Department of Endocrinology, The Affiliated Hospital of Southwest Medical University, Luzhou, Sichuan 646000, China; ^2^Collaborative Innovation Center for Prevention and Treatment of Cardiovascular Disease of Sichuan Province, Southwest Medical University, Luzhou, China; ^3^Department of Geratology, The People's Hospital of Leshan, Leshan, Sichuan 614000, China; ^4^Department of Nephrology, The People's Hospital of Leshan, Leshan, Sichuan 614000, China

## Abstract

**Background:**

Sumoylation is extensively involved in the regulation of NF-*κ*B signaling. PIASy, as a SUMO E3 ligase, has been proved to mediate sumoylation of I*κ*B kinase *γ* (IKK*γ*) and contribute to the activation of NF-*κ*B under genotoxic agent stimulation. However, the association of PIASy and NF-*κ*B signaling in the pathogenesis of diabetic nephropathy (DN) has not been defined.

**Methods:**

Rat glomerular mesangial cells (GMCs) were stimulated by high glucose; siRNA was constructed to silence the expression of PIASy; the expression of PIASy, SUMO isoforms (SUMO1, SUMO2/3), and NF-*κ*B signaling components was analyzed by Western blot; the interaction between IKK*γ* and SUMO proteins was detected by coimmunoprecipitation; and the release of inflammatory cytokines MCP-1 and IL-6 was assayed by ELISA.

**Results:**

High glucose significantly upregulated the expression of PIASy, SUMO1, and SUMO2/3 in a dose- and time-dependent manner (*P* < 0.05), induced the phosphorylation and sumoylation of IKK*γ* (*P* < 0.05), and then triggered NF-*κ*B signaling whereas MCP-1 and IL-6 were released from GMCs (*P* < 0.05). Moreover, these high glucose-induced effects were observably reversed by siRNA-mediated knockdown of PIASy (*P* < 0.05).

**Conclusion:**

The SUMO E3 ligase PIASy mediates high glucose-induced activation of NF-*κ*B inflammatory signaling, suggesting that PIASy may be a potential therapeutic target of DN.

## 1. Introduction

Extensive research shows that systemic and local low-grade inflammation and release of proinflammatory cytokines resulting from activation of nuclear factor-*κ*B (NF-*κ*B) signaling are involved in the development and progression of diabetes and diabetic nephropathy (DN) [1]. In fact, NF-*κ*B signaling plays critical roles in regulating immunity, cell survival, and expression of inflammatory cytokines. In the “resting” state, NF-*κ*B dimers are held inactive in the cytoplasm through association with I*κ*B proteins. Inducing stimuli triggers the activation of the I*κ*B kinase (IKK) complex, leading to phosphorylation, ubiquitination, and degradation of I*κ*B proteins. Released NF-*κ*B dimers translocate to the nucleus, bind to specific DNA sequences, and promote transcription of target genes. Thus, the core elements of the NF-*κ*B pathway are the IKK complex, I*κ*B proteins, and NF-*κ*B dimers [2].

Numerous regulatory posttranslational modifications (PTM), such as phosphorylation, acetylation, ubiquitination, and sumoylation, have been reported and shown to have effects on activating or inhibiting NF-*κ*B induced by diverse agents. Sumoylation is a process of PTM by a relatively small polypeptides called small ubiquitin-like modifier (SUMO) [3]. Sumoylation has been shown to regulate each of its targets in a specific way by altering its conformation, stability, or interaction and localization properties. Our previous study demonstrated that high glucose obviously induced the expression of SUMO isoforms (SUMO1, SUMO2/3) in GMCs, subsequently stimulated degradation of I*κ*B*α*, and triggered NF-*κ*B signaling by weakening the interaction between SUMO protein and I*κ*B*α* while promoting ubiquitination of I*κ*B*α* [4]. However, the association of SUMO ligases and NF-*κ*B signaling in DN has not been defined.

Sumoylation is a three-step process similar to ubiquitination that involves an E1-activating enzyme (Aos1/Uba2 heterodimer), an E2-conjugating enzyme (Ubc9), and E3 ligases, including the Ran-binding protein-2, Polycomb-2, and protein inhibitor of activated STAT (PIAS) proteins [5]. It is recently known that, in most cases, the final step of sumoylation reaction needs E3 ligases, which help to ensure substrate specificity and cell cycle dependence [6]. PIAS proteins were originally identified as repressors of the cytokine-induced STAT transcription factors. While PIAS proteins may act as E3 SUMO ligases, modulating the function of the target protein by adding SUMO tags, a growing body of evidence indicates that PIAS proteins may activate or repress transcription and play an important role in the regulation of transcription factors, including NF-*κ*B, Smads, and p53 [7]. The mammalian PIAS protein family contains five members: PIAS1, PIAS3, PIASx*α*, PIASx*β*, and PIASy; among PIAS proteins, PIASy can repress NF-*κ*B activity in mouse keratinocytes through interaction with the RelA/p65 subunit of NF-*κ*B, resulting in repressing the expression of CCL20 chemokine in response to TNF-*α* [8]. But the latest research proved that PIASy mediates IKK*γ* sumoylation and NF-*κ*B activation in response to oxidative stress conditions [9], while the roles of PIASy in regulating NF-*κ*B inflammatory signaling induced by high glucose is still unclear. In this study, we detected the changes of PIASy, SUMO1, and SUMO2/3; NF-*κ*B-related signaling molecules (I*κ*B*α*, p-I*κ*B*α*, p-IKK*γ*, IKK*γ*, NF-*κ*Bp65, and p-NF-*κ*Bp65); and downstream proinflammatory cytokines (MCP-1, IL-6) under high-glucose stress when the rGMCs were transfected with PIASy-siRNA or not, aiming to explore the roles of the SUMO E3 ligase PIASy on NF-*κ*B inflammatory signaling in the pathogenesis of DN.

## 2. Materials and Methods

### 2.1. Cell Culture and Treatment

Rat GMCs (HBZY-1) were purchased from the Fudan IBS Cell Center and were cultured in Dulbecco's modified Eagle medium (DMEM, Hyclone, USA) containing 5.6 mmol/L glucose and 10% fetal bovine serum (FBS, Bovogen, Australia) at 37°C and 5% CO_2_. GMCs were used for all experiments and were randomly divided into five groups: normal control group (NC group, with medium that contained 5.6 mmol/L glucose), 10 mmol/L glucose group (HG1 group, with medium that contained 10 mmol/L glucose), 20 mmol/L glucose group (HG2 group, with medium that contained 20 mmol/L glucose), 30 mmol/L glucose group (HG3 group, with medium that contained 30 mmol/L glucose), and osmotic pressure group (OP group, with medium that contained 5.6 mmol/L glucose + 24.6 mmol/L mannitol as a control). After cells in each group were induced for 6, 12, 24, 48, and 72 h, the culture supernatant was collected and the protein and mRNA were extracted for further study.

### 2.2. Small Interfering RNA Transfection

The PIASy duplex small interfering RNA (siRNA; RiboBio, China) was a pool of three-sequence siRNA targeting PIASy (number 1—sense: 5′-GCUGUAUGAGACUCGCUAUdTdT-3′ and antisense: 5′-AUAGCGAGCUCAUACAGCdTdT-3′; number 2—sense: 5′-GCAACUAUGGCAAGAGCUAdTdT-3′ and antisense: 5′-UAGCUCUUGCCAUAGUUGCdTdT-3′; and number 3—sense: 5′-GCAGCUUAUGACCAGCUCAdTdT-3′ and antisense: 5′-UGAGCUGGUCAUAAGCUGCdTdT-3′) or control siRNA (sense: 5′-UUCUCCGAACGUGUCACGU-3′ and anti-sense: 5′-ACGUGACACGUUCGGAGAA-3′). After the GMCs were transfected with PIASy-siRNA or control siRNA in serum-free Opti-MEM medium (Invitrogen, USA) with confluence of transfection (Roche) and siRNA at 37°C for 48 h, siRNA-mediated knockdown of PIASy cells was then stimulated by 5.6 mmol/L glucose or 30 mmol/L glucose. At the end of each experiment, the cells and culture supernatant were then collected for ELISA and Western blotting analysis.

### 2.3. Protein Extraction and Western Blotting

Total protein was extracted from GMCs using a protein extraction kit (Kaiji, China). Proteins were separated by sodium dodecyl sulfate-polyacrylamide gel electrophoresis (SDS-PAGE) and transferred to a polyvinylidene difluoride (PVDF) membrane (Millipore). Immunoblotting was performed using anti-PIASy antibody (mouse monoclonal antibody; 1 : 1000; Abcam, number ab211625), anti-SUMO1 antibody (rabbit monoclonal antibody; 1 : 800; Abcam, number 32058), anti-SUMO2/3 antibody (rabbit polyclonal antibody; 1 : 600; Millipore, number AB3876), anti-I*κ*B*α* antibody (mouse monoclonal antibody; 1 : 1000; CST, number 4814), anti-p-I*κ*B*α* antibody (ser32/36) (mouse monoclonal antibody; 1 : 1000; CST, number 9246), anti-NF-*κ*Bp65 antibody (rabbit polyclonal antibody; 1 : 1000; Beyotime, number AF0246), anti-p-NF-*κ*Bp65 antibody (ser536) (mouse monoclonal antibody; 1 : 2000; CST, number 13346), anti-p-IKK*γ* antibody (ser85) (rabbit polyclonal antibody; 1 : 1000; Bioworld, number BS4597), anti-IKK*γ* antibody (rabbit polyclonal antibody; 1 : 800; Santa Cruz, number sc-8830), and anti-*β*-actin antibody (mouse monoclonal antibody; 1 : 2000; Beyotime, number AF0003).

### 2.4. Enzyme-Linked Immunosorbent Assay (ELISA)

Rat MCP-1 and IL-6 secretion was measured using MCP-1 and IL-6 ELISA kits (Westang Bio-Tech, China) according to the manufacturer's protocols. MCP-1 and IL-6 protein levels were determined by comparing the samples to the standard curve generated by the kit.

### 2.5. Immunofluorescence

GMCs were grown on coverslips in 6-well plates. After overnight adherence, cells were incubated with 30 mmol/L high glucose or mannitol for 24 h as described above and then were fixed in 4% paraformaldehyde (Pierce Biotechnology, USA) and permeabilized in 0.25% Triton X-100 (Sigma, USA). Cells were blocked in 5% goat serum, followed by incubation with anti-PIASy and anti-SUMO1 or anti-SUMO2/3 antibody (dilution 1 : 100) overnight at 4°C. After washing, cells were incubated with rhodamine- and fluorescein isothiocyanate-conjugated secondary antibodies (Bio-Synthesis) for 45 min in the dark. 4′,6′-Diamino-2-phenylindole (DAPI) was used to stain the nucleus in the cells. The coverslips were washed and imaged with a DMIRE2 laser scanning confocal microscope (Leica, Germany). The values of semiquantitative analysis for average intensity were assessed by Image-Pro Plus 6.0 software.

### 2.6. Immunoprecipitation and Immunoblot Analysis

Approximately 24 h after being treated with media that contained high glucose and mannitol, the cells were harvested. Ice-cold immunoprecipitation lysis/wash buffer was added using a coimmunoprecipitation kit (Pierce Biotechnology, USA) with protease inhibitors (Roche, USA). The cell lysates were clarified by centrifugation at 13,000*g* for 10 min at 4°C, and the supernatants were subjected to immunoprecipitation. The supernatants were incubated with monoclonal anti-IKK*γ* antibody (rabbit polyclonal antibody; Santa Cruz, number sc-8830) and normal rabbit immunoglobulin G (IgG, Beyotime, China) for 12 h at 4°C. After incubation, protein A/G Sepharose was used for precipitation. The beads were washed with 1× conditioning buffer. The antigen-antibody complexes were collected, washed, and boiled in 2× lane marker nonreducing sample buffer. For the immunoblot analysis, proteins were probed with anti-SUMO1 antibody (rabbit monoclonal antibody; 1 : 800; Abcam, number 211625) or anti-SUMO2/3 antibody (rabbit polyclonal antibody; 1 : 600; Millipore, number AB3876).

### 2.7. Data Analysis

All data obtained from at least three independent experiments were expressed as the means ± standard deviation (SD), and between-group comparisons were analyzed using one-way analysis of variance (ANOVA), followed by the LSD post hoc test for multiple comparisons (SPSS 17 software). *P* < 0.05 was considered significant.

## 3. Results

### 3.1. High Concentrations of Glucose Induce PIASy and SUMO Isoform Expression and Colocalization in GMCs

**Figure 1:**
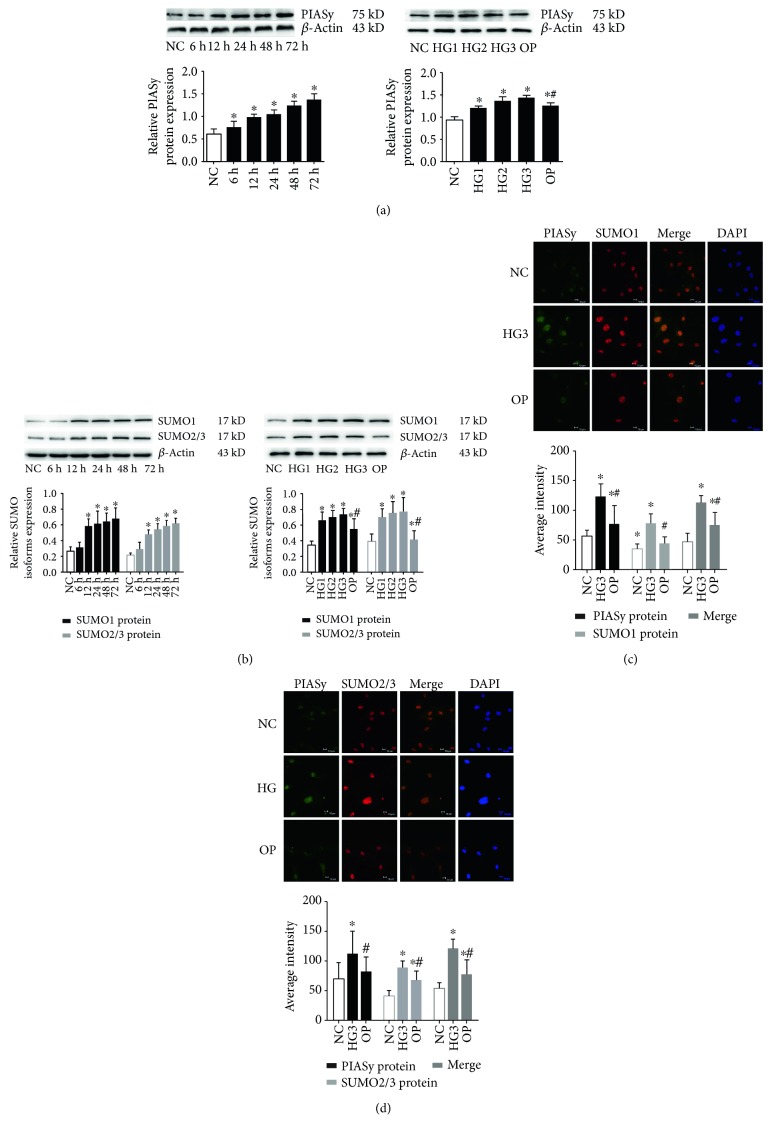
The expression of PIASy and SUMO isoforms (SUMO1, SUMO2/3) was induced by various times and various glucose concentrations. GMCs were treated with 30 mmol/L high glucose for 6, 12, 24, 48, and 72 h or the indicated concentrations of glucose or mannitol for 24 h; the protein expression of PIASy (a) and SUMO isoforms (SUMO1, SUMO2/3) (b) in lysates of cells was detected by Western blotting. Data were normalized with respect to *β*-actin and are expressed as mean ± SD (*n* = 5). The gray graphs confirmed these trends. Immunofluorescence was performed to determine intensity and subcellular localization of PIASy and SUMO1 (c) or SUMO2/3 (d) after GMC treatment with 30 mmol/L high glucose or mannitol for 24 h (400x). The merged images of PIASy and SUMO1 or SUMO2/3 stainings in each group were shown, and DAPI was used to stain the nucleus in the cells. The values of semiquantitative analysis for average intensity were assessed, and the gray graphs confirmed these trends. ^∗^*P* < 0.05 compared with the NC group, ^#^*P* < 0.05 compared with the 30 mmol/L high glucose stimulation group.

Compared to the NC group, the expression of PIASy was significantly induced following 6, 12, 24, 48, and 72 h of exposure to 30 mmol/L glucose (*P* < 0.05); the highest relative expression of PIASy protein was observed after stimulation of 72 h. Moreover, PIASy protein levels were also significantly enhanced by different concentrations of glucose at 24 h (*P* < 0.05); the highest relative expression of PIASy was detected in the 30 mmol/L glucose group. A significant difference was also found between the OP group and NC group. However, PIASy protein levels were significantly decreased in the OP group compared with the 20 mmol/L and 30 mmol/L glucose groups (*P* < 0.05) (Figure 1(a)), suggesting that high glucose concentration increased the expression of PIASy in a time- and dose-dependent manner, and osmotic pressure had a little effect on the high glucose-induced PIASy expression. Consistent with PIASy, the expression of SUMO isoforms (SUMO1 and SUMO2/3) was significantly increased by high glucose in a time- and dose-dependent manner (*P* < 0.05) (Figure 1(b)). Moreover, the merged images of immunofluorescence showed that PIASy colocalized with SUMO1 (Figure 1(c)) or SUMO2/3 (Figure 1(d)) in the nucleus, compared to the NC group and OP group; the average intensity of these proteins was strongly enhanced in the 30 mmol/L glucose group (*P* < 0.05). These results suggested that high glucose increased the expression of PIASy, SUMO1, and SUMO2/3, while inducing the colocalization of PIASy and SUMO1 or SUMO2/3 in the nucleus of GMCs.

### 3.2. High Glucose Induced the Phosphorylation and Sumoylation of IKK*γ* in GMCs

**Figure 2:**
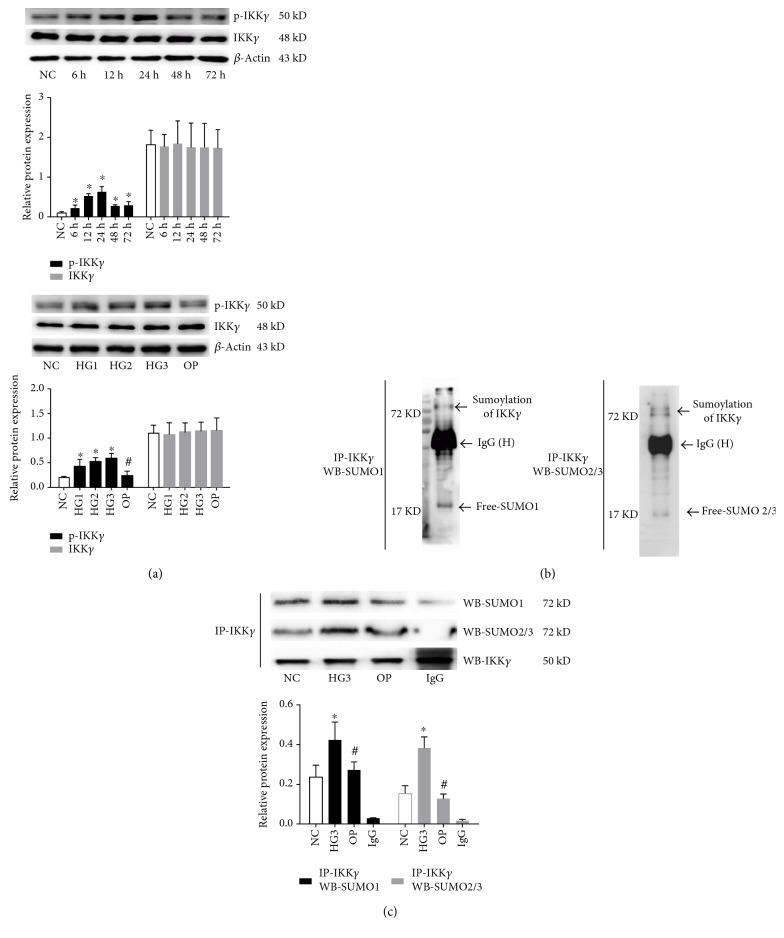
The phosphorylation and sumoylation of IKK*γ* were induced by high glucose. (a) The protein expression of p-IKK*γ* and IKK*γ* in lysates of GMCs was detected after high glucose challenge for various times and various glucose concentrations by Western blotting. (b) IKK*γ* sumoylation was detected by immunoprecipitation (IP) with anti-IKK*γ* antibody followed by Western blotting with anti-SUMO1 or anti-SUMO2/3 antibody. IKK*γ* was conjugated with SUMO in GMCs. IgG (H) marks the IgG heavy chain. (c) GMCs were treated with 30 mmol/L high glucose or mannitol for 24 h. Anti-IKK*γ* immunoprecipitates were subjected to immunoblotting with anti-SUMO1 or anti-SUMO2/3 antibody to detect IKK*γ* and SUMO-IKK*γ* proteins; normal IgG antibody was used as a negative control. IKK*γ* that was sumoylated by SUMO1 or SUMO2/3 was induced by high glucose. The gray graphs confirmed these trends. Data are expressed as mean ± SD (*n* = 3). ^∗^*P* < 0.05 compared with the NC group, ^#^*P* < 0.05 compared with the 30 mmol/L high glucose stimulation group.

Compared to the NC group, there was no significant change in the expression of IKK*γ* following different concentrations of high-glucose treatment, while high glucose, especially 30 mmol/L glucose, observably induced the expression of phosphorylation of IKK*γ* after 24 h stimulation (*P* < 0.05) (Figure 2(a)). Next, we performed the immunoprecipitation and immunoblot analysis to determine whether SUMO is involved in IKK*γ* sumoylation in GMCs. As shown in Figure 2(b), IKK*γ* antibodies immunoprecipitated a polypeptide of about 72 kD that was recognized by the SUMO-specific antibody. The results showed that SUMO1 and SUMO2/3 were coimmunoprecipitated with IKK*γ*, and the SUMO-induced modification of IKK*γ* was detected on endogenously expressed proteins, suggesting that SUMO and IKK*γ* were able to form a complex in GMCs. To determine whether IKK*γ* sumoylation is affected by high glucose, we assessed the effect of 30 mmol/L glucose and mannitol treatment on IKK*γ* sumoylation. Interestingly, the interaction between IKK*γ* and SUMO1 or SUMO2/3 was increased by high glucose (*P* < 0.05) (Figure 2(c)), but osmotic pressure had a little effect on the high glucose-induced IKK*γ* sumoylation. Taken together, these data indicated that phosphorylation and sumoylation of IKK*γ* were induced by high glucose in GMCs.

### 3.3. High Glucose Significantly Activated NF-*κ*B Inflammatory Signaling by Degradation of I*κ*B*α*

**Figure 3:**
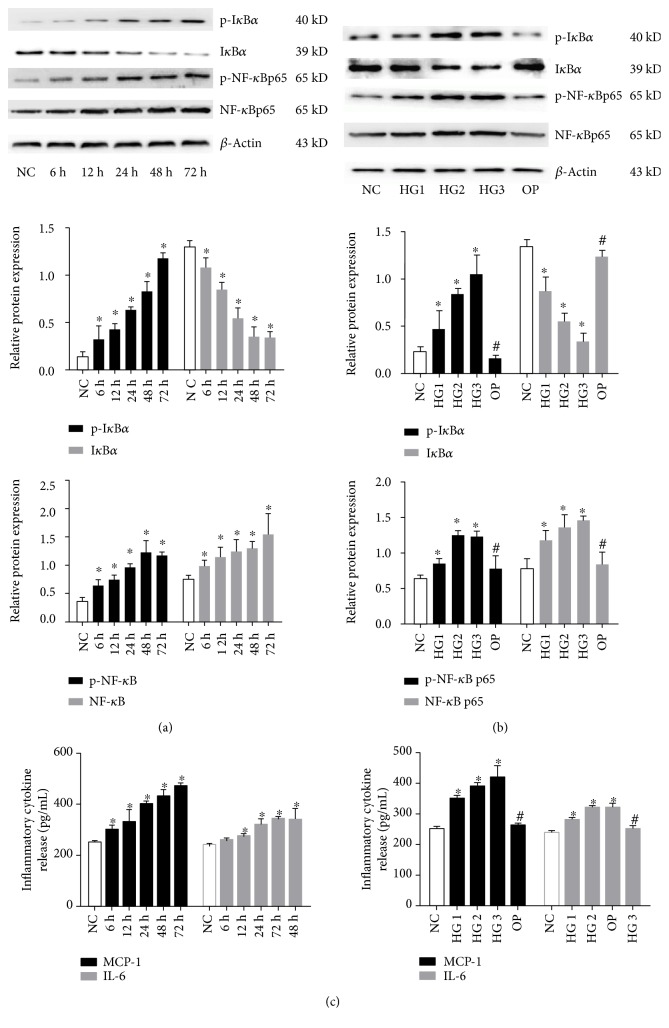
High glucose significantly activated the NF-*κ*B inflammatory signaling by degradation of I*κ*B*α*. GMCs were treated with 30 mmol/L high glucose for 6, 12, 24, 48, and 72 h (a) or the indicated concentrations of glucose or mannitol (b) for 24 h; the expression of p-I*κ*B*α*, I*κ*B*α*, p-NF-*κ*B, and NF-*κ*B in lysates of cells was detected by Western blotting. Data were normalized with respect to *β*-actin and are expressed as mean ± SD (*n* = 3). The gray graphs confirmed these trends. MCP-1 and IL-6 protein levels in the cell culture supernatant were determined by ELISA-based quantification (c). ^∗^*P* < 0.05 compared with the NC group, ^#^*P* < 0.05 compared with the 30 mmol/L high glucose stimulation group.

The relative expression of p-I*κ*B*α*, p-NF-*κ*Bp65, and NF-*κ*Bp65 was markedly increased following 6, 12, 24, 48, and 72 h of exposure to 30 mmol/L glucose (*P* < 0.05) (Figure 3(a)). Furthermore, p-I*κ*B*α*, p-NF-*κ*Bp65, and NF-*κ*Bp65 were also significantly induced by different concentrations of high glucose, especially in the 30 mmol/L glucose group (*P* < 0.05); the addition of 30 mmol/L mannitol to normal glucose did not change the expression of these proteins (*P* > 0.05), indicating that the high glucose-induced activation of NF-*κ*B signaling molecules was not an osmotic effect (Figure 3(b)). However, the expression of I*κ*B*α* was significantly attenuated by high glucose in a time- and dose-dependent manner (*P* < 0.05) (Figures 3(a) and 3(b)). Consistent with the activation of signaling molecules, the release of inflammatory cytokines MCP-1 and IL-6 from GMCs was also obviously increased by high glucose in a time- and dose-dependent manner (*P* < 0.05) (Figure 3(c)). These data suggested that high glucose, but not osmotic pressure, induced the degradation of I*κ*B*α* and activated NF-*κ*B inflammatory signaling.

### 3.4. High Glucose-Induced Sumoylation of IKK*γ* and NF-*κ*B Activation Were Significantly Reversed by siRNA-Mediated Knockdown of PIASy

**Figure 4:**
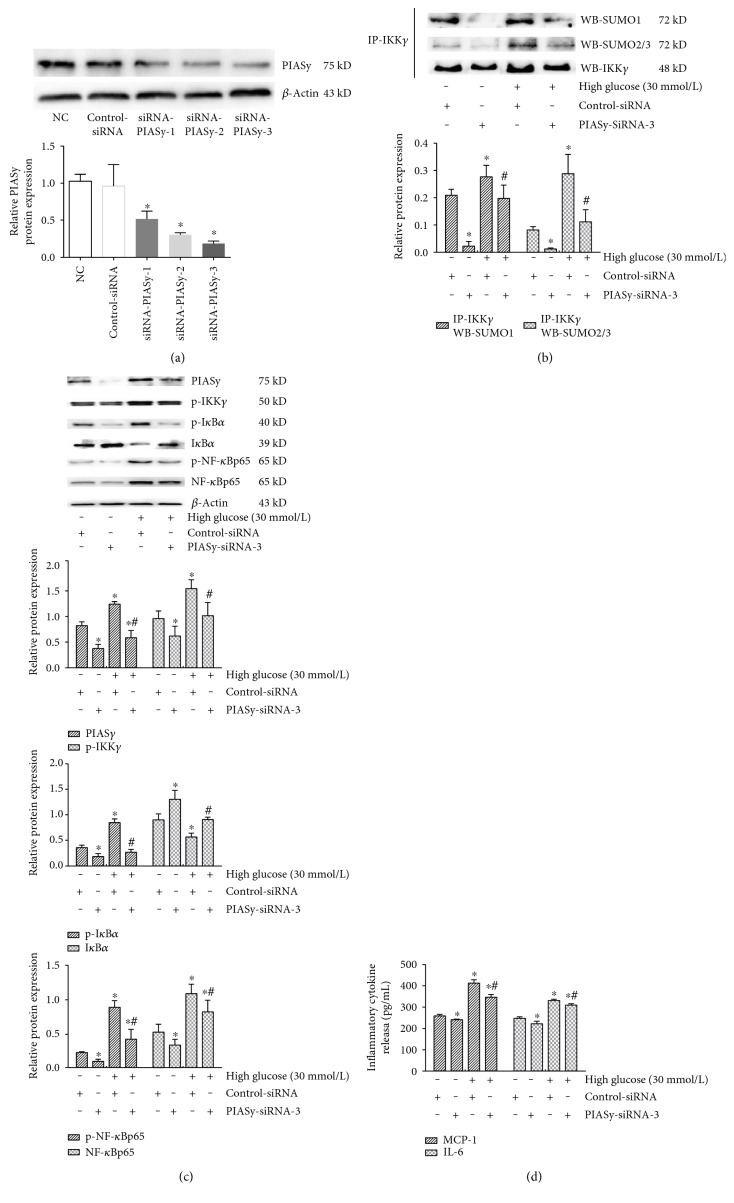
High glucose-mediated activation of NF-*κ*B inflammatory signaling was reversed by siRNA-PIASy. (a) Western blotting analysis of protein expression after siRNA-mediated knockdown of PIASy has proven effective in silencing target genes of PIASy by siRNA-PIASy-3. (b) Immunoprecipitation and immunoblot analysis of the interaction between IKK*γ* and SUMO1 or SUMO2/3 induced by high glucose was reversed by siRNA-PIASy-3. (c) Western blotting analysis showed that the changes in PIASy, p-IKK*γ*, p-I*κ*B*α*, I*κ*B*α*, p-NF-*κ*Bp65, and NF-*κ*Bp65 expression after high glucose challenge were significantly reversed by siRNA-PIASy-3. (d) ELISA-based quantification indicated that high glucose-induced release of MCP-1 and IL-6 from GMCs was blunted by siRNA-PIASy-3. The results are presented as mean ± SD (*n* = 5); the gray graphs confirmed these trends. ^∗^*P* < 0.05 compared with the NC group, ^#^*P* < 0.05 compared with the 30 mmol/L high glucose stimulation group.

PIASy expression was successfully inhibited by PIASy-siRNA (*P* < 0.05), especially by PIASy-siRNA-3, which was chosen for subsequent experiments (Figure 4(a)). The interaction between IKK*γ* and SUMO1 or SUMO2/3 induced by high glucose was reversed by siRNA-PIASy-3 (Figure 4(b)). Indeed, siRNA-mediated knockdown of PIASy also markedly attenuated the phosphorylation of IKK*γ* and I*κ*B*α*, inhibited the degradation of I*κ*B*α*, and reversed the activation of NF-*κ*B induced by 30 mmol/L glucose for 24 h (*P* < 0.05) (Figure 4(c)). To investigate whether the downstream proinflammatory cytokines of NF-*κ*B signaling may be inhibited by siRNA-mediated knockdown of PIASy, as expected, elevated MCP-1 and IL-6 release from GMCs was blunted by PIASy gene silencing (*P* < 0.05) (Figure 4(d)). These results suggested that siRNA-mediated knockdown of PIASy inhibited the sumoylation of IKK*γ* and the activation of NF-*κ*B inflammatory signaling; in other words, the SUMO E3 ligase PIASy plays an important role in the activation of NF-*κ*B signaling induced by high glucose.

## 4. Discussion

GMC proliferation and hypertrophy, ECM accumulation, and consequent renal fibrosis induced by high glucose, AGEs, or LPS have been recognized as major pathogenic events in the progression of renal failure in DN [10]. Numerous evidences demonstrate that the activation of NF-*κ*B in GMCs plays a central regulatory role in the expression of various inflammatory cytokines (such as MCP-1 and IL-6) involved in the occurrences of DN [11]. In agreement with previous reports, our present results indicate that high glucose may be involved in the pathogenesis of DN via manifesting upregulation of phosphorylation of I*κ*B*α*, which results in I*κ*B*α* degradation and activation of NF-*κ*B. Our experimental results also showed that the release of MCP-1 and IL-6 from GMCs was significantly upregulated by high glucose in a dose-dependent and time-dependent manner, suggesting that high glucose induces a renal inflammatory state. Therefore, novel therapeutic strategies that target gene-special regulators of NF-*κ*B may prove to be more efficient for the treatment of DN.

Sumoylation plays an important role in multiple biological processes, such as protein interactions, protein stability, nuclear-cytoplasmic trafficking, transcriptional regulation, DNA repair, and cellular signaling pathways [12, 13]. Multiple signal transduction molecules of the NF-*κ*B pathway, such as I*κ*B*α*, IKK, RelA, and P100, can be modified by SUMO [8, 9, 14]. SUMO1 can mediate the sumoylation of I*κ*B*α*, resulting in sustaining the stability of I*κ*B*α*, in case of degradation, and inhibiting inflammatory NF-*κ*B pathway activation [15]. Overexpression of SUMO4 also contributes to enhancing the sumoylation of I*κ*B*α* and regulating NF-*κ*B activation under external stimulus stimulation, which is considered to be strongly associated with type 1 diabetes [16]. SUMO E3 ligases play a key role in sumoylation and ensure substrate specificity and cell cycle dependence in response to different stresses. Previous studies reported that PIASy is located predominantly in the nucleus and interacts with various transcription factors; however, it has also been reported that PIASy interacts with cytoplasmic proteins, such as axin [17]. Studies showed that PIASy cooperated with PIAS1 to regulate the specificity and magnitude of NF-*κ*B-mediated gene activation [18]. Consistent with previous results, our data showed that high glucose increased the protein expression of SUMO1 and SUMO2/3 in a time- and dose-dependent manner; furthermore, high glucose also upregulated the expression of PIASy and induced the colocalization of PIASy and SUMO1 or SUMO2/3 in the nucleus of GMCs; osmotic stress had a little effect on the expression of SUMO and PIASy proteins, suggesting that PIASy, SUMO1, and SUMO2/3, which may be considered cellular stress proteins, may play an important role in the regulation of the NF-*κ*B pathway in response to high glucose during renal injury.

Under oxidative stress, PIASy mediates transglutaminase (TG2) sumoylation and inhibits TG2 ubiquitination and proteasome degradation, leading to sustenance of TG2 activation, which prevents I*κ*B*α* sumoylation and results in NF-*κ*B activation and an uncontrolled inflammatory response, but SUMO1 or PIASy gene silencing can induce TG2 degradation and restore I*κ*B*α* sumoylation, thus switching off inflammation [19]. Our previous studies have already demonstrated that high glucose decreased sumoylation of I*κ*B*α* by weakening the interaction between SUMO2/3 and I*κ*B*α*, suggesting that the stability of SUMO-I*κ*B*α* plays a predominance function in regulating NF-*κ*B inflammatory signaling in response to high glucose [4]. Here, our present results suggested that high glucose-induced upregulation of PIASy could affect the stability of I*κ*B*α*, leading to the degradation of I*κ*B*α* and activation of NF-*κ*B inflammatory signaling, but the underlying mechanisms are yet unidentified.

IKK*γ*, known as NEMO (NF-*κ*B essential modulator), a noncatalytic subunit of the IKK complex, plays an essential regulatory role for the NF-*κ*B canonical pathway. A series of publications now provide information about ubiquitination, sumoylation, or phosphorylation of IKK*γ* which regulates its function in the IKK complex. These modifications might also regulate a cytosolic pool of free IKK*γ* that controls the activation of NF-*κ*B induced by genotoxic stress [20, 21]. In this study, we performed an experiment to determine whether high glucose is involved in IKK*γ* sumoylation in GMCs. Our data showed that no significant change in the protein expression of IKK*γ* was found in GMCs induced by high glucose and high osmotic pressure, while the expression of p-IKK*γ* and the interaction between IKK*γ* and SUMO1 or SUMO2/3 were observably induced by high glucose, suggesting that high glucose was involved in phosphorylation and sumoylation of IKK*γ*. However, as a SUMO ligase, the role of PIASy in this process needs to be further confirmed.

Previous research revealed that PIASy preferentially stimulates site-selective modification of IKK*γ* by SUMO1, but not SUMO2 and SUMO3, *in vitro*. PIASy-IKK*γ* interaction is increased by genotoxic stress and oxidative stress; siRNA-PIASy inhibits IKK*γ* sumoylation and NF-*κ*B activation, and overexpression of PIASy enhances these events [9]. Further studies have found that activated poly(ADP-ribose)-polymerase-1 (PARP-1) and a PAR-binding motifs (PARBM) in PIASy are required to trigger IKK*γ* sumoylation, which in turn permits IKK and NF-*κ*B activation, as well as NF-*κ*B-regulated resistance to apoptosis [22], and exported sumoylated IKK*γ* acts as a substrate. IKK*γ* monoubiquitination is a prerequisite for genotoxic IKK and NF-*κ*B activation but also promotes cytokine signaling [23]. However, whether PIASy participates in high glucose-induced NF-*κ*B inflammatory signaling in GMCs has not been defined. Here, our results firstly demonstrate that high glucose-induced phosphorylation and sumoylation of IKK*γ* were reversed by siRNA-PIASy. Moreover, degradation of I*κ*B*α*, activation of NF-*κ*B, and release of downstream inflammatory cytokines MCP-1 and IL-6 from GMCs induced by high glucose were blunted by siRNA-PIASy. Based on our findings, we suggest a new model for the activation of NF-*κ*B inflammatory signaling: exposure of the GMCs to high glucose results in the overexpression of the SUMO E3 ligase PIASy and SUMO proteins, which causes the SUMO proteins to bind to IKK*γ*, mediating the phosphorylation and sumoylation of IKK*γ*; the subsequent degradation of I*κ*B*α* and activation of NF-*κ*B in turn result in the processing of MCP-1 and IL-6 release from GMCs, eventually promoting the renal low-grade inflammation. In other words, these combined results firstly reveal that upregulation of PIASy may play an important role in NF-*κ*B activation in the pathogenesis of DN. However, our studies by no means rule out other potential mechanisms by which sumoylation may regulate the NF-*κ*B pathway, in view of the fact that the regulatory mechanisms of NF-*κ*B are extremely complex and the types of SUMO E3 ligases are diverse.

In summary, our study has firstly demonstrated that high glucose increased the expression of PIASy in a dose- and time-dependent manner, subsequently induced the phosphorylation and sumoylation of IKK*γ* and then degradation of I*κ*B*α*, and activated the NF-*κ*B inflammatory signaling in GMCs, which can be switched off by siRNA-mediated knockdown of PIASy. The present results support the hypothesis that the SUMO E3 ligase PIASy mediates high glucose-induced activation of NF-*κ*B inflammatory signaling, suggesting that PIASy may be a potential therapeutic target of DN.
